# Supplementary material for: HIVprotI: an integrated web based platform for prediction and design of HIV proteins inhibitors
Source: J Cheminform. 2018 Mar 9;10:12. doi: 10.1186/s13321-018-0266-y (PMC5845081; doi:10.1186/s13321-018-0266-y)
Supplement: Supplementary file 2 — Additional file 2. Source code of HIVProtI web server. [file 13321_2018_266_MOESM2_ESM.zip › HIVprotI_Source-code/hivprotI/jsmol/jsv_predict.htm]

JSpecView/JSME/nmrdb/NIH-Resolver demo


|  |  |  |
| --- | --- | --- |
|  |  | info clear spectrum (note that OH and NH hydrogens will not be shown)  This page illustrates how we can use JSME (the JavaScript Molecular Editor)  along with JSpecView to quickly get a simulated spectrum for a compound of our choice.  It loads slowly because it is a test page; it will load much faster when not in testing.   JSmol on this page calls servers in Frederick, Maryland (NIH resolver, for name-to-structure)  and Lausanne, Switzerland (nmrdb, for structure-to-spectrum)   Draw a chemical structure  or search for a chemical identifier such as **caffeine** or *CCOCC*,  then press .   Note that these spectra are *just predictions.*  They may differ significantly from actual NMR spectra. |
| credits: JSmol and page development: Bob Hanson JSME: Peter Ertl, Bruno Bienfait JSpecView: Robert Lancashire, Bob Hanson nmrdb: Luc Patiny NIH Resolver: Markus Sitzmann Java2Script: Zhou Renjian |  |  |
